# Supplementary material for: Comparison of Clinical and Imaginal Features According to the Pathological Grades of Dysplasia in Branch-Duct Intraductal Papillary Mucinous Neoplasm (BD-IPMN) for Personalized Medicine
Source: J Pers Med. 2023 Jan 12;13(1):149. doi: 10.3390/jpm13010149 (PMC9863534; doi:10.3390/jpm13010149)
Supplement: Supplementary file 1 [file jpm-13-00149-s001.zip › jpm-2100594-supplementary.pdf]

Supplementary Table S1. Summary of national guidelines.

	Type	Surgical indication	EUS FNA indication
2015 AGA		Solid component and dilated pancreatic duct and/or concerning features on EUS FNA (solid component and a dilated pancreatic duct)	At least 2 high risk features<br>Size ≥3 cm<br>Dilated main pancreatic duct<br>Presence of an solid component
2016 JAMA		Symptomatic<br>MD-IPMN or Mixed type<br>Cysts with High risk features<br>MPD>10 mm<br>Solid component<br>Mural nodule<br>High risk features on EUS FNA<br>Positive cytology<br>Mural nodule<br>Thickened cyst wall<br>MPD 5-9 mm<br>Cyst ≥3 cm	No High risk feature, but ≥3cm

2017 Revisions of international consensus Fukuoka,	MD<br>BD	MPD >10 mm<br>Jaundice<br>Mural nodule	
	BD	WF with concerning features on EUS FNA<br><br>WF<br>Pancreatitis<br>Cyst≥3 cm<br>Enhancing mural nodule<5mm<br>Thickened/enhaincg cyst walls<br>MPD 5-9 mm<br>Abrupt change in pancreatic duct<br>Lymphadenopathy<br>Increased CA19-9<br>Cyst growth rate≥5 mm/2years<br><br>EUS FNA<br>Cytology	Presence of WF

		Mural nodule ≥5mm<br>MPD involvement<br>anyone of thickened walls, intraductal mucin or mural nodules	
2018 EEBGPCN	MD	Recommendation for the surgery	
	BD	Absolute<br>Positive cytology<br>Solid mass<br>Jaundice<br>Enhancing mural nodule≥5 mm<br>MPD≥10 mm<br>Relative<br>Cyst growth rate≥5 mm/1years<br>Increased CA19-9 (≥37U/ml)<br>MPD 5-9 mm<br>Cyst≥4 cm<br>New onset DM<br>Acute pancreatitis	Either clinical or radiological features of pancreas cystic neoplasm, in cases of unclear diagnosis based on cross-sectional imaging

		Enhancing mural nodule <5 mm	
